# Nano/Micro-Structural Supramolecular Biopolymers: Innovative Networks with the Boundless Potential in Sustainable Agriculture

**DOI:** 10.1007/s40820-024-01348-x

**Published:** 2024-03-08

**Authors:** Roohallah Saberi Riseh, Mohadeseh Hassanisaadi, Masoumeh Vatankhah, Rajender S. Varma, Vijay Kumar Thakur

**Affiliations:** 1 Department of Plant Protection, Faculty of Agriculture, Vali-e-Asr University of Rafsanjan, Imam Khomeini Square, Rafsanjan, 7718897111 Iran; 2 Centre of Excellence for Research in Sustainable Chemistry, Department of Chemistry, Federal University of São Carlos, São Carlos, SP 13565-905 Brazil; 3 Biorefining and Advanced Materials Research Center, Scotland’s Rural Collage (SRUC), Edinburgh, EH9 3JG UK

**Keywords:** Supramolecular, Biopolymers, Sustainable agriculture, Nanotechnology

## Abstract

Sustainable agriculture plays a crucial role in meeting the growing global demand for food while minimizing adverse environmental impacts from the overuse of synthetic pesticides and conventional fertilizers. In this context, renewable biopolymers being more sustainable offer a viable solution to improve agricultural sustainability and production. Nano/micro-structural supramolecular biopolymers are among these innovative biopolymers that are much sought after for their unique features. These biomaterials have complex hierarchical structures, great stability, adjustable mechanical strength, stimuli-responsiveness, and self-healing attributes. Functional molecules may be added to their flexible structure, for enabling novel agricultural uses. This overview scrutinizes how nano/micro-structural supramolecular biopolymers may radically alter farming practices and solve lingering problems in agricultural sector namely improve agricultural production, soil health, and resource efficiency. Controlled bioactive ingredient released from biopolymers allows the tailored administration of agrochemicals, bioactive agents, and biostimulators as they enhance nutrient absorption, moisture retention, and root growth. Nano/micro-structural supramolecular biopolymers may protect crops by appending antimicrobials and biosensing entities while their eco-friendliness supports sustainable agriculture. Despite their potential, further studies are warranted to understand and optimize their usage in agricultural domain. This effort seeks to bridge the knowledge gap by investigating their applications, challenges, and future prospects in the agricultural sector. Through experimental investigations and theoretical modeling, this overview aims to provide valuable insights into the practical implementation and optimization of supramolecular biopolymers in sustainable agriculture, ultimately contributing to the development of innovative and eco-friendly solutions to enhance agricultural productivity while minimizing environmental impact.

## Highlights

- Safer and biodegradable nano/micro-supramolecular biopolymers transform farming practices.
- Supramolecular biopolymers improve soil structure, water retention, and plant nutrient uptake.
- Encapsulation of bioactive compounds and their controlled release by nano/micro-supramolecular biopolymers enables targeted delivery.
- Biopolymer networks can potentially be embedded in biosensors for crop protection goals.

## Graphical abstract

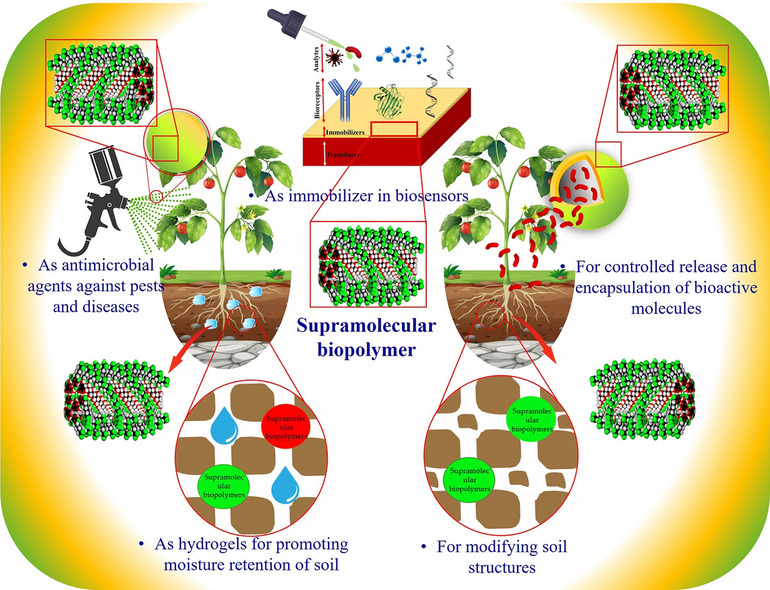

## Introduction

The requisite for sustainable agriculture is driven by several issues, such as growing populations, finite resources, and environmental concerns. The use of agrochemicals, excessive water consumption, and soil deterioration are common in traditional farming methods, which harm ecosystems and human health [1]. Sustainable agriculture is an ecological approach to food production that aims to minimize environmental impacts, conserve natural resources, and protect biodiversity for the long term [2, 3]; it ensures food security, reduces the effects of climate change, and promotes a healthier ecosystem [4, 5]. Consistent with the ideals of sustainable agriculture, ongoing efforts are being made to investigate inventive approaches that can improve the sustainability and productivity of farming methods. An emerging alternative that has garnered considerable interest in recent years is the utilization of biopolymers in the field of agriculture [6], mainly owing to their capacity to augment sustainability and productivity as they offer a more sustainable approach to agriculture [7, 8]. They are derived from renewable resources such as plants [7, 9, 10], microbes [11], animals [12–14], sea sources [15, 16], or biomass [17] and are biodegradable, thus minimizing their environmental effects. Biopolymers can reduce reliance on non-renewable resources, reduce pollution from chemical fertilizers and pesticides, and minimize the soil erosion and nutrient runoff [18–20]. Emerging from ongoing scientific developments in biopolymer research, a novel category of biopolymers known as nano/micro-structural supramolecular biopolymers has been developed.

The advent of nano/micro-structural supramolecular biopolymers has significantly transformed several domains, presenting unparalleled prospects for their advanced applications. They refer to a class of biomaterials composed of complex, hierarchical structures at the nano- and micro-scale levels. These novel biopolymers possess unique qualities compared to regular polymers, notably in terms of their natural propensity for self-assembly, a trait that results in highly organized structures, providing increased stability. Additionally, these biopolymers can engage in reversible supramolecular interactions, enabling dynamic behaviors like self-repair and responsiveness to external stimuli. Their inherent flexibility allows them to function effectively in various scenarios, making them valuable in controlled release systems and drug delivery platforms. Compared to standard biopolymers, their enhanced biodegradability holds remarkable significance in several appliances, particularly in controlled release systems [21, 22].

This new generation of biopolymers is formed through non-covalent interactions, such as hydrogen, chalcogen, halogen bonding, electrostatic interactions, and hydrophobic interactions, enabling intricate network assembly [23–25]. These supramolecular biopolymers exhibit unique properties, including high stability, tunable mechanical strength, stimuli-responsiveness, ease of functionalization, and self-healing [26, 27]. Their structural versatility allows the incorporation of various functional molecules, such as enzymes, growth factors, and antimicrobial agents, further expanding their applications [28]. Supramolecular biopolymers are increasingly recognized as promising biomaterials for a wide range of applications, including but not limited to biomedical and agricultural fields [29–31]. Despite the aforementioned advantages, they encounter several complexities, and constraints, a significant obstacle being their evaluation procedure. Evaluating the aggregation mechanism in supramolecular polymerization is an intricate process, as the concentration of the oligomers can significantly impact the extent of aggregation [32] thus necessitating meticulous management and measurement. The biological properties of certain biopolymers, such as proteins, rely on 3D third-dimensional structures [33]. These exceptional and dynamic structures are achieved through carefully designed folding processes of polypeptide chains encoded by sequences, with the help of various noncovalent connections within the chains. However, replicating this folding process to create higher-order structures is extremely difficult for supramolecular polymers. In order to synthesize such copolymers, it is necessary to develop appropriate methods for combining chemically different monomers that can generate distinct domains. Due to the inherent tendency of these monomers to separate themselves, construction of these unique supramolecular block copolymers poses a challenge [34]. Notwithstanding these obstacles, the domain of supramolecular biopolymers is growing, and scientists are investigating novel approaches to surmount these constraints and create materials with enhanced functionality.

**Fig. 1:**
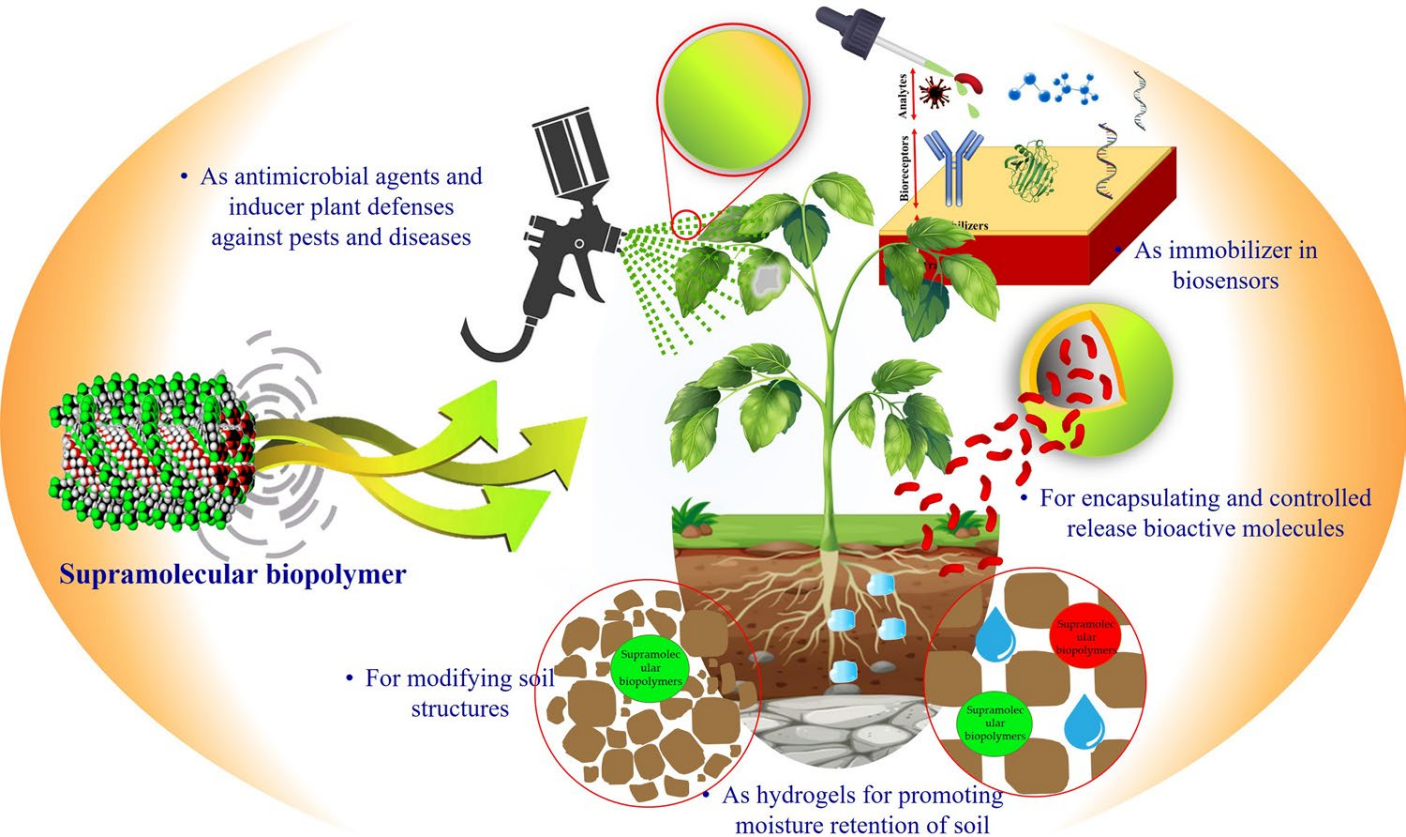
The potential of supramolecular biopolymers for enhancing sustainable agriculture

The agricultural industry is currently facing significant challenges. However, the distinctive characteristics and potential of nano/micro-structural supramolecular biopolymers offer a promising opportunity to modernize the approach and practice of agriculture. Indeed, the potential of nano/micro-structural supramolecular biopolymers in agriculture can be a great transformation as these innovative networks hold promise for addressing several challenges the agricultural industry presently encounters. They can radically reform farming practices, enhance crop productivity, improve soil health, and contribute to sustainable agriculture. These systems enhance nutrient absorption, nutrient utilization, and soil structure, promoting moisture retention, aeration, root development, water use efficiency, and reduced irrigation needs [35–37]. These biopolymers can enhance plant defenses against pests and diseases by incorporating antimicrobial agents and immune-stimulating compounds, thus reducing reliance on chemical pesticides [38, 39]. The use of these novel biopolymer networks in crop protection is also noteworthy in view of their potential utilization as biosensors; they can be used as coatings on transducers, influence the immobilization and stabilization of biological elements and reduce non-specific interactions with the sample matrix [40]. Moreover, these biopolymers are in line with sustainable agriculture practices as they minimize environmental impact, promote resource efficiency, and serve as eco-friendly alternatives to synthetic materials [41]. Figure 1 depicts the potential of supramolecular biopolymers for enhancing sustainable agricultural.

Notwithstanding the demonstrated significant promise in profoundly altering agriculture and addressing critical complications, there is dearth of additional understanding regarding their precise uses and tactics for optimization. Although the current body of literature mostly centers on synthesis and characterization of supramolecular biopolymers and their utilization in medicine, there is a noticeable paucity of research examining their potential applications in the agricultural sector. Hence, due to the lack of extensive study on the specific applications of these unique generations of biopolymers in the context of various farming practices, it is imperative to address this knowledge gap to promote sustainable agriculture by examining their specific uses. The present overview initially presents fundamental scientific knowledge about supramolecular biopolymers, highlighting them distinctively from conventional polymers, elucidating their structural characteristics, and exploring their applications in the development of hydrogels with emphasis centering around utilization in agriculture. Furthermore, this analysis investigates the potential of nano/micro-structural supramolecular biopolymers as biosensors in crop protection applications, including challenges and future prospects for their deployment in agriculture. Through experimental investigations and theoretical modeling, this enquiry provides valuable insights into the practical implementation and optimization of supramolecular biopolymers in sustainable agriculture. The outcomes will help contribute to the development of innovative and eco-friendly solutions for enhancing agricultural productivity while minimizing the environmental impact.

## How do Supramolecular Biopolymers Differ from Conventional Polymers?

**Fig. 2:**
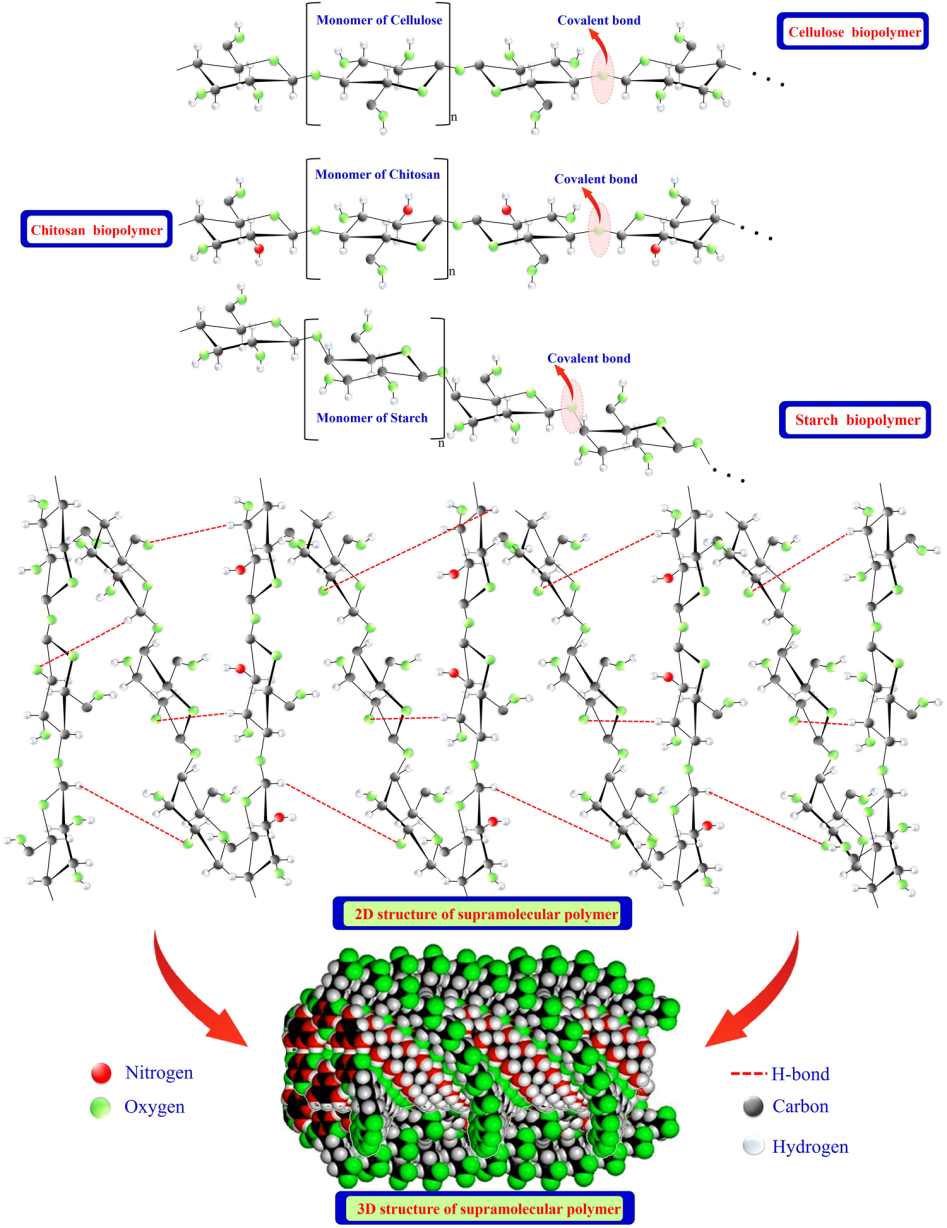
Covalent and hydrogen bonds that illustration the difference between conventional polymers and supramolecular biopolymers

Supramolecular biopolymers differ from conventional polymers in their molecular structure and behavior (Fig. 2). Conventional polymers typically comprise covalently bonded repeating units, forming long, chain-like structures and encompassing fixed molecular weights and well-defined chemical compositions [42]. On the other hand, supramolecular biopolymers are formed through non-covalent interactions, such as hydrogen bonding, $\pi -\pi$ stacking, metal–ligand coordination, host–guest interactions and hydrophobic interactions [43], which allow them to self-assemble into higher-order structures, such as helices, fibers, or gels; proteins, nucleic acids, and polysaccharides are examples of supramolecular biopolymers [26]. They often display dynamic and reversible behavior, as the non-covalent interactions can be disrupted or reestablished under appropriate conditions. This flexibility endows new biopolymer materials with many attractive properties, including ease of synthesis and functionalization, structurally responsive nature, and the possibility of incorporating an array of assorted ligands via co-assembly of the building blocks [44]. In contrast, conventional polymers have more rigid structures and limited ability to external cues or self-repair [45]. It can be concluded that supramolecular polymers offer three main advantages. Firstly, the reversibility of their non-covalent interactions allows for easy modification and recovery of the polymer. They also exhibit a wide range of response capabilities to different stimuli. Secondly, supramolecular biopolymers have superior environmental adaptability and resilience compared to other polymer types. Furthermore, research has discovered that supramolecular polymers can engage in covalent crosslinking to create shape-memory materials. Thirdly, the formation process of supramolecular polymers in a water system indicates that the primary factors responsible for their formation are hydrophobic, electrostatic, and ionic-dipole interactions [46].

## Supramolecular Structures

The supramolecular structure of biopolymers, such as proteins, nucleic acids, and polysaccharides, is crucial for their properties and functions. For instance, proteins are composed of amino acid building blocks and exhibit several levels of supramolecular organization. The primary structure represents the linear sequence of amino acids, while the secondary structure involves the local folding patterns, such as alpha-helices and beta-sheets; hydrogen bonds and other non-covalent interactions stabilize these secondary structures. The tertiary structure refers to the spatial arrangement of the secondary structure elements, and the quaternary structure describes the assembly of multiple protein subunits into a fully functional protein complex. Through these hierarchical arrangements, proteins acquire their specific shapes and functions [47] and similarly nucleic acids also possess a supramolecular structure; DNA, the genetic material, adopts a double-helical structure formed by the complementary base pairing between nucleotides. Hydrogen bonding between adenine, thymine, cytosine, and guanine stabilizes this double helix structure. The twisted arrangement of the two DNA strands allows for efficient packaging and accessibility of genetic information during processes like DNA replication and transcription [48]. Polysaccharides, like cellulose and chitin, are additional examples of biopolymers encompassing supramolecular structures. Cellulose, for instance, forms strong fibers due to extensive hydrogen bonding between individual cellulose chains. These intermolecular interactions form a highly organized and rigid structure, contributing to the strength and rigidity of plant cell walls [49]. Thus, the supramolecular structure of biopolymers contributes to their unique properties and functionalities, including enzymatic activity, information storage, structural support, and recognition capabilities. Understanding and manipulating these supramolecular structures are essential in various fields, including biotechnology, material science, medical science, and agriculture.

Supramolecular biopolymers, including polysaccharides, exhibit a diverse binding mode facilitated by non-covalent interactions [50]. Polysaccharides, known for their remarkable biological and biomedical applications, are an exciting class of molecules [51, 52]. Within these polymers, inter- and intra-chin interactions along with ion pairs, give rise to primary, secondary, tertiary, and quaternary structures, resulting in supramolecular architectures. These architectures serve as a foundation for various applications, including agrochemical delivery, to enhance the stability and availability of poorly soluble fertilizers and pesticides [53]. Supramolecular chemistry mainly relies on non-covalent interactions such as metal–ligand coordination, hydrogen bonds, hydrophobic, and Van der Waals forces. These interactions are essential in controlling self-assembly processes, including those involved in biological systems. Moreover, they serve as a design principle for developing innovative products tailored for agricultural and biomedical applications [54]. In the context of supramolecular biopolymers, hydrophobized polysaccharides have been synthesized to create supramolecular structures in aqueous environments. Their interactions with soluble proteins and other molecular assemblies, such as liposomes, oil-in-water emulsions, black lipid membranes, and monolayers, have been extensively studied [55]. These investigations aim to understand and harness the unique binding modes of supramolecular biopolymers, leading to advancements in fields like agrochemical delivery of nanopesticides and nanofertilizers.

## Supramolecular Polymers as Hydrogels

Supramolecular hydrogels, also called physical hydrogels, are created through reversible, non-covalent interactions between macromolecules such as biopolymers and low-molecular-weight gelators [43]. Typically, supramolecular hydrogels develop in two steps: self-assembly and crosslinking. The formation of hydrogen bonds along with other interactions such as host-guest, hydrophobic, coordination, and electrostatic attractions contributes to the stabilization conferred by this structure [56]. The process of gel formation can be initiated by a range of chemical (pH alteration), physical (light, temperature, magnetic field, and ultrasound) factors, and enzymatic reactions [57]. Supramolecular polymers as hydrogels offer several benefits. One remarkable benefit of these structures is their capacity to self-repair and regain their original characteristics (self-healing property) by creating reversible non-covalent interactions [58]. Moreover, their inherent biocompatibility and biodegradability make them attractive for biomedical and agricultural applications. Improving the gelation process provides precise control over the hydrogels’ mechanical properties, porosity, water retention, and release kinetics, tailoring them to specific requirements [59]. Furthermore, supramolecular hydrogels possess a distinctive property of undergoing a reversible sol–gel transition in response to stimuli relevant to biological systems. This unique property makes them particularly valuable for the preparation of injectable hydrogels, enabling precise delivery of bioactive compounds [43]. This versatility allows hydrogels to be applied in diverse areas such as biomedical applications, medicine, tissue engineering, environmental engineering, and agriculture [60]. Nano-scale hydrogels or nanogels based on supramolecular biopolymers have gained significant recognition and attention in agriculture due to their various beneficial features and potential applications, including large surface area, high water retention capacity, controlled and targeted delivery of agrochemicals and bioactive agents [61, 62]. Additionally, their nano-scale properties enable them to penetrate complex plant tissues, exerting their benefits at the cellular level [63].

Research in supramolecular hydrogels is advancing rapidly, with ongoing efforts to explore new design strategies, optimizing their properties, and broaden their application. By harnessing the power of non-covalent interactions, supramolecular polymers hold great promise as hydrogels, paving the way for innovative solutions in agricultural science and beyond.

## Supramolecular Liquid Crystals

In recent decades, liquid-crystalline polymers, often known as LC polymers, have garnered much attention. The well-designed LC polymer structures can contribute to a wide range of disciplines, including new electronics, energy, environment, resources, and biotechnologies [64, 65] as their novel developments have brought attention to their prospective deployment in various fields. The term “liquid crystal” (LC) describes a phase transition between a solid with a highly organized crystal structure and an isotropic liquid; accordingly, they are commonly known as intermediate phases (or mesophases) [66]. Liquid crystals, with their hierarchical molecular arrangement or ability to self-assemble, offer a foundation for the creation of a wide range of nanostructured materials [67, 68]. In addition, LC-based biosensors are extensively used in diagnostics [69] as they can potentially be incorporated in the design biosensors for the diagnosis of phytopathogens. Additionally, the self-assembly and molecular arrangement of LCs are appealing for creating materials that can respond to stimuli. In these materials, modifications in the nanostructure can lead to changes in attributes such as form, color, or porosity. Therefore, LC polymers have been prominent in the field of sustainable agriculture for encapsulating bioactive substances, being considered smart and functional materials in view of their ability to respond to stimuli features [70–73].

Furthermore, there are recently developed functional LC polymers alongside the formation of covalently linked traditional LC polymers. Supramolecular hydrogen-bonded liquid crystal (LC) polymers have been developed by using supramolecular entities as exemplified by Kato and Frechet who initially synthesized these polymers by creating complementary hydrogen bonds [74]. Due to their selectivity, directionality, and dynamic nature, hydrogen bonds are perfect noncovalent interactions for the fabrication of supramolecular materials. Hydrogen-bonding interactions play a crucial role in directing the arrangement of molecules in LC materials and improving the durability of polymers. They can also serve as a key factor in bestowing materials the capacity to respond to stimuli [75].

Initially, supramolecular hydrogen-bonded LC polymers were synthesized by linking side-chain of LC polymers with stilbazoles via the benzoic acid groups [74]. Furthermore, Kato et al. [76] and van Nunen et al. [77] described the creation of host–guest liquid crystal supramolecular polymers by forming inclusion compounds.

Thus, it can be construed that the organized molecular structure or self-assembly in supramolecular LCs offers a foundation for creating diverse nanostructured materials. These materials have potential applications in agriculture, including controlled release systems, intelligent sensors for detecting plant pathogens, and carriers for agricultural chemicals. Interestingly, supramolecular LCs possess a dynamic nature and exhibit responsiveness to environmental changes, a trait that renders them highly suitable for various advanced agriculture appliances. These applications comprise the creation of stimuli-responsive and self-healing materials for agricultural infrastructure, such as encapsulation goals, as well as the controlled release of fertilizers and pesticides.

## Difference Between Nano- and Micro-Supramolecular Polymers

Nano- and micro-supramolecular polymers are both fascinating fields of study in material science and chemistry, as they differ in size and properties. Micro-supramolecular polymers are larger structures, formed by self-assembling larger macromolecules or by aggregating smaller supramolecular units. They exhibit higher mechanical strength and larger sizes than nano-supramolecular polymers, making them more suitable for applications requiring bulk material or macroscopic structures. On the other hand, nano-supramolecular polymers refer to structures on nano-scale, typically ranging from 1 to 100 nm, and comprise smaller molecular units or building blocks that assemble into ordered arrangements through nan-covalent interactions [78]. Nano-supramolecular polymers often possess unique properties such as high surface area, enhanced stability, and tailored functionalities, making them suitable to be applied in agriculture as innovative delivery systems [79]. Under specific conditions, micro-supramolecular polymers can undergo a remarkable transformation, transitioning into nano-supramolecular structures. Factors such as alterations in solvent characteristics, temperature variation, concentration adjustment, or the application of external stimuli drive this metamorphosis. These diverse factors play integral roles in orchestrating the shift from micro- to nano-structures, unraveling unique possibilities in the realm of supramolecular chemistry [80, 81]. It should be considered that depending on the system and the nature of the supramolecular structures involved, the specific conditions required for this transformation may vary.

## Potential of Nano/Micro-Structural Supramolecular Biopolymers in Sustainable Farming Agriculture

Nano/micro-structural supramolecular biopolymers have tremendous potential in transforming agriculture, offering a range of applications that can enhance crop protection, improve soil health, and mitigate environmental health. Their non-toxicity and biodegradability render these novel materials appealing to modernized agriculture with improved sustainability in farming. Additionally, their versatility makes them central players in defining the future of farming [82, 83]. First, these advanced materials act as a matrix for developing controlled-release agrochemicals, ensuring a steady and targeted nutrient supply to plants over an extended period, minimizing waste, and reducing frequency use and environmental impact [20]. They can be exploited to develop smart nanomaterials that encapsulate and deliver agrichemicals such as pesticides, herbicides, and fungicides directly to targeted plant tissues. Moreover, applying nano/microstructural supramolecular for stimuli-responsive encapsulation of agrochemicals introduces an innovative approach to agriculture. These biopolymers have the ability to respond to specific triggers such as pH, temperature, moisture rate, and enzymes, improving their efficiency and minimizing the risk of overdosing and underdosing [84]. These smart carrier systems also open up new possibilities to increase the efficiency and effectiveness of various agricultural agents, including biological control agents, other bioactive agents, and plant stimulants [85, 86]. Secondly, incorporating these biopolymers into soil amendments enhance soil structure and fertility due to superior water retention, promoting nutrient availability and preventing soil erosion [18, 87]. A critical role of nano/micro-structural supramolecular biopolymers is their application in soil improvement and nutrient management. By forming stable nano/micro-networks, they improve soil aggregation, which promotes better root penetration, aeration, and moisture retention [88]. They can act as carriers for fertilizers and slow-release agents, improving nutrient efficiency and reducing potential environmental impacts such as nutrient volatilization and leaching [89]. In sustainable agriculture, water scarcity is a significant concern. In the context of water management strategies, these superabsorbent structures enhance water retention in the soil, particularly in arid and drought-prone regions, thus reducing irrigation requirements and enhancing water-use efficiency [90]. Thirdly, coating seeds with supramolecular biopolymers protects against biotic and abiotic stresses. Slow-release coatings also offer a sustainable release of nutrients, pesticides, and growth-promoting components, providing better germination and seedling growth [91]. These structures also contribute to developing eco-friendly and biodegradable packaging materials that can potentially replace conventional plastic packaging and decrease the environmental contamination [92]. By utilizing renewable resources, they promote a more sustainable strategy in the agricultural industry. For example, Ma et al. [93] developed a hydroxypropyl cellulose-modified Uio-66 for pyraclostrobin delivery wherein the system exhibited a good biological safety, and did not have any detrimental effect on the soil microbial community, normal growth of the target rice crop and non-target organisms especially *Daphnia magna*, was observed with a toxicity of 4.6 times lower than that of PYR-TC after 48 h.

By exploiting the application potential of nano/micro-structural supramolecular biopolymers, agriculture can embrace sustainability and efficient practices while reducing the environmental impact. Table 1 summarized a variety of supramolecular biopolymers, their formulation, and targeted appliances.

**Table 1:** Several types of synthesized supramolecular biopolymers, their formulation, and targeted applications

Supramolecular biopolymer type	Formulation	Application(s)	Refs
Hydroxypropyl cellulose-modified Uio-66	Microsphere	Delivery system, fungicidal activity	[93]
Supramolecular microgel based on chitosan, salicylaldehyde, and urea	Hydrogel	Increasing water holding capacity	[94]
Supramolecular polymer based on chitosan, gelatin, β-cyclodextrin, and Arabic gum loaded with ferrous sulfate	Microsphere	Increasing water retention capacity, controlled-release of actives, antibacterial activity, heavy metal ions adsorption	[95]
Supramolecular polymer based on gelatin, sodium alginate, and pickling zeolite	Microsphere	Encapsulation goals, increasing water retention, controlled-release of actives	[96]
Supramolecular polymer based on guar gum with acrylic acid and crosslinking with ethylene glycol di methacrylic acid	Hydrogels	Enhancing moisture retention of soil	[97]
Supramolecular polymer based on bacterial cellulose	Hydrogels	Irrigation applications	[98]
Supramolecular polymer based on chitosan, polyvinyl alcohol and lignin nanoparticles	Microsphere	Controlled-release of actives	[99]
Supramolecular polymer based on cationic starch and chitosan	Microcapsules	Encapsulation goals	[100]
Supramolecular polymer based on soy whey protein and pyridine-grafted poly (hydroxyethyl methacrylate)	Microsphere	Encapsulation goals	[39]
Supramolecular polymer based on pectin on porous hollow silica microcapsules loaded with prochloraz	Microcapsules	Fungicidal activity	[101]
Supramolecular polymer based on alginate carboxyl, and the amino residues of chitosan	Microcapsules	Delivery system	[102]
Supramolecular polymer based on chitosan nano-humic acid-curcumin	Microcapsules	Antibacterial and antioxidant agent	[103]
Supramolecular polymer based on sodium alginate and poly(diallyl dimethyl ammonium chloride)	Biosensors	Diagnosis	[104]
Supramolecular polymer/Co^2+^	Biosensors	Immobilizing glucose oxidase for enzymatic glucose sensing	[105]

### Supramolecular Biopolymers for Improving Soil Structure and Moisture Retention

Nano/micro- supramolecular polymers have the potential to enhance soil structure and improve moisture retention. Due to their unique properties, these polymers can facilitate the formation of a well-structured soil matrix, promoting better water infiltration and reducing the risk of soil erosion [90]. Moreover, the nano-scale dimension of these polymers allows them to effectively bind soil particles together, forming aggregates that enhance soil stability. This property improves the soil structure, promotes moisture retention and enhances the nutrient availability and root development, thus contributing to better plant growth and increasing agricultural productivity [106]. On the other hand, nano/micro-supramolecular polymers possess superabsorbent capability, absorbing and retaining large amounts of water several times higher than their own weight. Superabsorbent have remarkable adsorption characteristics, enabling them to bind and collect specific molecules or contaminants efficiently. These materials, such as activated carbon, zeolites, or silica gel, are frequently manufactured synthetically [107–109]. High-adsorbent materials have significant adsorption capability due to their expanded surface area and pore geometries. On the other hand, biopolymers, are obtained from organic origins from substances like chitosan [110, 111], alginate [110], cellulose [111], and proteins such as gelatin [110]. They are acknowledged for their ability to interact well with living organisms, their capability to break down naturally, and their capacity to be maintained over time [112]. Biopolymers are utilized in controlled drug release, wastewater treatment, and delivery systems for active components in many industries. In terms of the release strategy, superabsorbent can be used to absorb and retain molecules or compounds, preventing their discharge into the environment [17]. Biopolymers can be deployed as matrices or carriers in controlled release systems to encapsulate active compounds and aid in their release [113–115]. Highly adsorbent materials mostly utilize adsorption or physical interaction for retaining chemicals, whilst biopolymers commonly employ diffusion or degradation processes to achieve regulated release. The specific material and application determine the variation in the release mechanism [17]. Superabsorbent has a greater adsorption capacity because of their optimized architectures. Hence, by integrating with superabsorbent, biopolymers can be customized and modified to improve their adsorption characteristics for targeted contaminants. For instance, chitosan, a biopolymer derived from chitin, effectively adsorbs heavy metals and dyes from wastewater [116]. Furthermore, biopolymeric adsorbent systems, such as chitosan/cellulosic materials, have demonstrated high efficiency in the biosorption of industrial textile effluents [117]. While high-adsorbent materials are well-established for their adsorption capabilities, biopolymers offer a sustainable and versatile alternative with promising adsorption performance, especially in environmental remediation and sustainable agricultural practices.

**Fig. 3:**
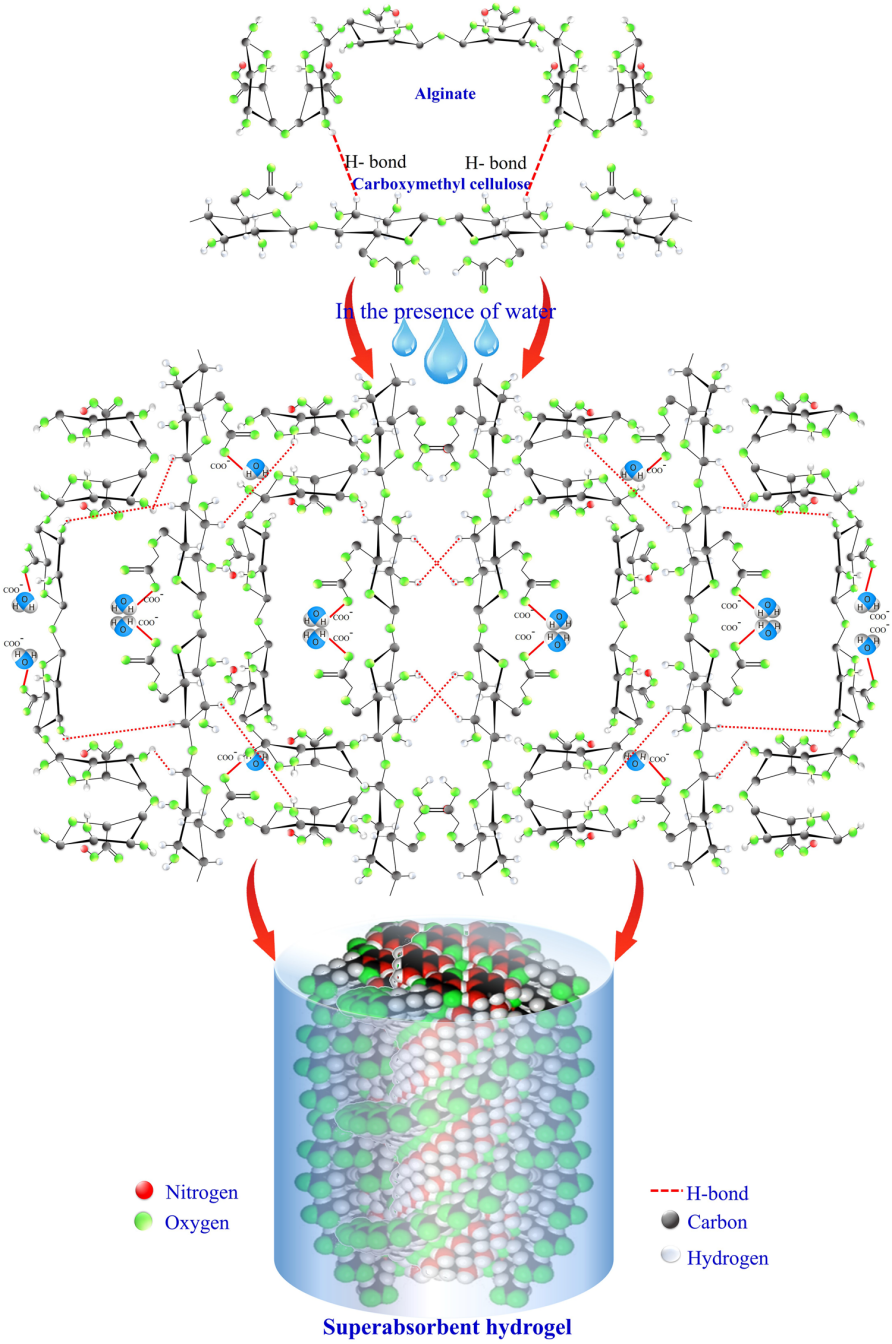
The mechanism for water absorption by supramolecular hydrogels

Under soil conditions, these nano/micro-gels act as reservoirs, storing water during periods of excess and gradually releasing it during water insufficiency. Accordingly, this superabsorbent property significantly enhances soil water-holding capacity, reducing irrigation frequency and conserving water resources [118]. For example, supramolecular microgel based on chitosan, salicylaldehyde, and urea have demonstrated impressive water absorption of 68 g g^−1^, resulting in a notable upsurge in soil water holding capacity by 154%. Moreover, this formulation nearly doubled the nitrogen content in the soil, resulting in approximately 70% higher plant growth compared to the bulk soil [94]. Li et al. [95] fabricated a multifunctional microsphere supramolecular soil conditioner based on chitosan, gelatin, β-cyclodextrin, and Arabic gum loaded with ferrous sulfate. This formulation exhibited thermal decomposition temperature, high water retention capacity, controlled-release behavior, antibacterial performance, and heavy metal ions adsorption, making it ideal for improving soil quality. An unusual type of supramolecular microsphere was prepared through physical crosslinking of gelatin, sodium alginate, and pickling zeolite loaded with FeO(OH); they encapsulated urea, exhibiting high swelling, water retention, and sustained-release properties [96]. This innovative formulation acts as an effective soil conditioner, enhancing soil quality and plant growth. Novel superabsorbent hydrogels have been fabricated by grafting guar gum with acrylic acid and crosslinking with ethylene glycol di methacrylic acid. These hydrogels significantly improve several soils’ physiochemical properties, including enhancing moisture retention capacity by up to 54%, and increasing its porosity by up to 9% compared to the original soil composition [97]. A supramolecular composite hydrogel, consisting of a double crosslinked interpenetrating network within a porous matrix of bacterial cellulose, was employed for monitoring irrigation applications. This hydrogel exhibited exceptional mechanical properties and showcased distinctive fluorescence capabilities. It retained a high level of strength even after undergoing repeated cycles of swelling and drying, as well as enduring long-term compressive processes. Additionally, the hydrogel demonstrated a non-conventional fluorescence behavior, which remained stable even under extreme environmental conditions like high/low temperatures and exposure to various organic solvents [98]. Besides their direct impact, nano/micro- supramolecular biopolymers can indirectly affect soil structure by promoting favorable conditions for soil microorganisms. By increasing moisture retention, these structures support the proliferation of microbial populations, leading to a potential increase in soil microbial community and their ability to function effectively [119]. A thriving microbial community can further enhance soil structure by producing extracellular substances such as polysaccharides and glues, acting as natural adhesives, binding soil particles together, and improving soil stability [120]. For instance, Ramachandran et al. [121], found that in situ bacterial dextran produced by *Leucononstoc mesenteroids*, demonstrated impressive efficiency in soil stabilization with comparable mechanical properties as commercial polymers. Applying xanthan gum biopolymer as a soil amendment improved particle binding in sandy soil and improved water uptake efficiency under drought conditions [122]. This, in turn, contributes to better water infiltration, reduced soil erosion, and enhanced nutrient availability for plants. Therefore, while micro/nano-supramolecular biopolymers do not directly increase soil microbial population, their positive impact on soil moisture can create conditions supporting soil microorganisms’ growth and activity. Figure 3 depicts the water-absorbing mechanism of supramolecular hydrogels.

### Supramolecular Biopolymers for Enhancing Nutrient Absorption

**Fig. 4:**
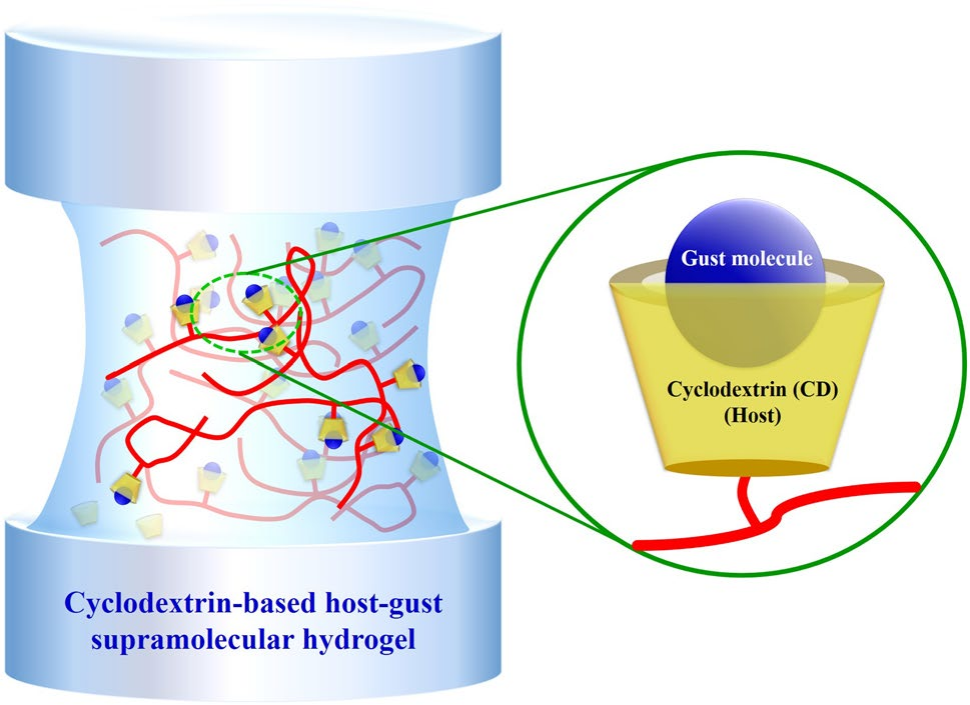
Supramolecular hydrogel based on cyclodextrin host–guest interaction

The prospective application of nano/micro-structural supramolecular biopolymers in enhancing nutrient absorption and crop utilization is promising. These advanced biopolymers, with their intricate structures at nano- and micro-levels, offer several advantages in improving plant nutrient uptake and utilization. The unique properties of supramolecular biopolymers make them ideal for acting as stimuli-responsive carriers, providing controlled and targeted release of macro- and micro-nutrients, ensuring a more efficient delivery to plant roots. This can improve nutrient absorption and plant utilization [123, 124]. Moreover, these biopolymers can encapsulate and protect nutrients from degradation or leaching in the soil, thereby enhancing their stability and availability over a more extended period [125]. The superabsorbent capability of these biopolymers positively influences nutrient absorption and utilization in plants by maintaining optimal soil moisture levels; this property ensures a consistent water supply, promotes efficient nutrient uptake, facilitates nutrient transport within the plant, and buffer against extreme moisture fluctuations [126, 127]. Furthermore, these biopolymers promote the development of robust root systems by offering a favorable growth environment, which enables plants to explore a large soil volume for extracting nutrients, leading to better nutrient absorption and utilization. Host–guest supramolecular hydrogels based on cyclodextrin have gained significant promise in drug delivery as they exhibit shear-thinning behavior, possess stimuli-responsive properties, and have remarkable biocompatibility [128]. The structure of host–guest supramolecular hydrogels based on cyclodextrin is depicted in Fig. 4. As a result, they are not limited to drug delivery applications alone but also hold great potential for the intelligent delivery and controlled release of agrochemicals.

The construction of a self-assembly structure based on silk fibroin (SF) and tannic acid (TA) as a biocompatible molecular glue has been investigated. The gelation of SF with TA was achieved through hydrophobic interactions, $\pi -\pi$ stacking, and hydrogen bonding. This sol–gel transition of SF occurs under physiological conditions (pH 7.4, 37 °C), which created a supramolecular assembly for loading elements like Zn and Fe [124, 129]. Loading urea granules using a supramolecular structure composed of chitosan/polyvinyl alcohol and lignin nanoparticles resulted in excellent slow-release nitrogen properties over a period of time. The outcomes revealed that addition of lignin nanoparticles was advantageous as it significantly extended the lifespan of the coated urea fertilizer, reaching up to 18 days compared to 5 days of unmodified chitosan/polyvinyl alcohol [99]. Foliar application of supramolecular humic acid modified with monocalcium caused an increase of about 30% in all growth and yield parameters of cauliflower in calcareous soils [130]. Lima-Tenório et al. [100] encapsulated *Azospirillum Brasilense*, a biofertilizer, using supramolecular microbeads based on cationic starch and chitosan physically crosslinked with sodium tripolyphosphate. The remarkable outcome was that these microbeads not only maintained the viability of S. *brasilense* for at least 60 days, but also improved maize chlorophyll content, shoot fresh weight, and root length. Although the application of these biopolymers in agriculture is still being researched and developed, their potential for enhancing nutrient utilization in plants holds great promise for sustainable and efficient agricultural practices.

### Supramolecular Biopolymers for Protecting Plants Against Biotic and Abiotic Stresses

**Fig. 5:**
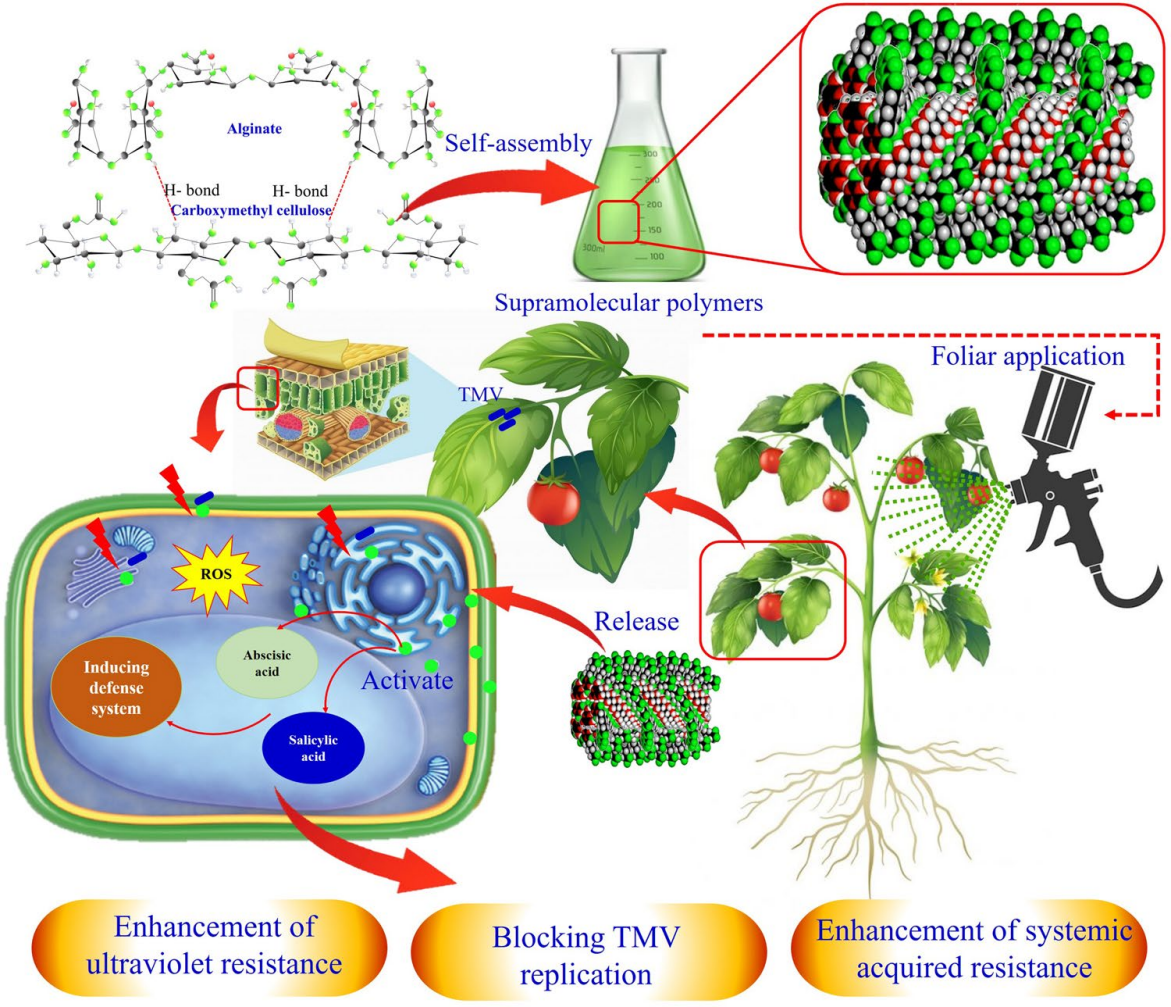
Mechanisms for supramolecular polymers against biotic and abiotic stresses

Supramolecular biopolymers, with their unique micro- and nano-scale structures, can enhance plant systems’ resilience and immune responses in the face of biotic stresses. These structures stimulate defense compounds and cell walls, activating specific signaling pathways against pathogens and pests [131]. Chitosan’s strong impact on plant immune response may be due to its interaction with acidic pectin in the plant cell wall, which can bind to calcium and form chain dimers. This interaction alters the structure of pectin and pectin dimers, triggering an alarm signal about cell wall degradation and pathogen presence. The plant reacts to chitosan–pectin dimer complexes significantly stronger than the individual components [132]. In addition to their potential as plant protectors, nano/micro-structural supramolecular biopolymers serve as carriers for pesticides, biological control agents, antimicrobial natural compounds, and biostimulators. These structures improve pest and disease management efficiency by allowing controlled release and targeted delivery of active substances, as encapsulated agents are released gradually and precisely at the desired site [133]. Supramolecular hydrophobic modified agar exhibited significant promise in effectively encapsulating curcumin and achieving controlled release specifically under weak alkaline conditions [134]. This finding highlights the potential application of these hydrogel microspheres in delivering hydrophobic pesticides for agricultural purposes. In a recent study, Xiang et al. [39] introduced a novel nanocarrier composed of soy whey protein and pyridine-grafted poly (hydroxyethyl methacrylate). This supramolecular nanocarrier was designed to encapsulate a potential antiviral candidate agent called Bingqingxiao (BQX). Under in vivo conditions, the formulated BQX@PP@S NPs (nanoparticles) demonstrated a protective activity 1.4 times higher than BQX alone against the tobacco mosaic virus. Moreover, when the BQX@PP@S NPs were applied to plants' leaves, they activated host plant defense responses by upregulating the expression of genes associated with salicylic acid (SA) and abscisic acid (ABA). This finding suggests that the BQX@PP@S NPs exhibit antiviral activity and serve as plant nutrition, leading to a considerable increase in crops’ fresh and dry weight by 24.7%, and 19.9%, respectively. Figure 5 depicts the mechanisms of supramolecular polymers against biotic and abiotic stresses.

A novel approach was employed to achieve smart delivery of chlorpyrifos using supramolecular nanoplatforms composed of alginate and (Ca^2+^)-ethylenediamine tetraacetate. These halloysite nanocarriers demonstrated dual pH responsiveness, extending their control efficiency, and enhancing their resistance to ultraviolet irradiation and high temperatures. Additionally, the strong interaction between alginate and plant leaves significantly improved the foliar adhesion property of HCECA (halloysite, Ca^2+^, EDTA^2−^, chlorpyrifos, and alginate) against rinsing by the rain. Compared to pristine chlorpyrifos and HCECA (the control pesticide without alginate), the foliar adhesion property of HCECA was strengthened by 86% and 45%, respectively. This enhancement highlights the potential of using these nanocarriers to mitigate the impact of rain rinsing on pesticide effectiveness [135]. In a recent study [93], researchers have designed a remarkable method for delivering pyraclostrobin using a hydroxypropyl cellulose-modified Uio-66 framework. The investigation demonstrated that this system displayed exceptional release behavior of PYR when exposed to acidic and cellulose-rich environments, mimicking the conditions during *Rhizoctonia solani* infestation. Moreover, this innovative formulation exhibited significantly higher fungicidal activity compared to commercially available microencapsulated formulations. Yang et al. [101] fabricated a highly innovative pH and pectinase dual responsive smart delivery system of pesticide by incorporating pectin on porous hollow silica microcapsules loaded with prochloraz by electrostatic adsorption. Notably, these microcapsules exhibited a long-term control efficiency against *Sclerotinia sclerotiorum* without compromising rapeseed plant growth. The implementation of this controlled-release vehicle also resulted in a significant reduction in pesticide usage. Moreover, this core–shell structure successfully enhanced the stability of prochloraz, preventing its degradation under alkaline and light conditions while concurrently minimizing its detrimental impact on the aquatic environment. Similarly, the supramolecular self-assembly of pectin with calcium carbonate (CaCO_3_)_,_ precipitated on the surface of prochloraz ionic liquid micelles, led to the development of double-shelled microcapsules as a green delivery system for pesticides. The control efficacy of these dual pH and pectinase-responsive microcapsules against *S*. *sclerotiorum* was 1.65 times higher compared to PR emulsion in water at the same concentration. Furthermore, the photostability of these microcapsules surpassed that of PRO EW by 1.58 times, indicating their superior performance [136]. In a study by Singh et al. [102], the physical and chemical properties of alginate beads were improved by the electrostatic interaction of their carboxyl residues and the amino components of chitosan. This supramolecular polymer, combined with cenosphere, was employed as a delivery system for imidacloprid, providing a controlled and gradual release behavior in response to various pH conditions, replicating different soil types. Moreover, it demonstrated twice the reduced leaching of the pesticide compared to commercially available formulations. Hydrogen-bonding interactions initiated supramolecular chitosan nano-humic acid-curcumin nano/micro-coatings with distinctive mechanical properties as well as antibacterial and antioxidant activities. They can be applied to control the long-term release of natural preservatives onto fruit surfaces. Applying this coating significantly decreased the fruit decay, resulting in an extension of the shelf-life of perishable fruits by at least 9 days compared to uncoated samples [103].

Regarding abiotic stresses, supramolecular biopolymers have the potential to function as protective barriers, safeguarding plants against detrimental environmental conditions, such as drought, excessive temperature, or high salinity. These structures offer a range of benefits by retaining moisture, regulating temperature, or scavenging reactive oxygen species, ultimately mitigating plant stress. However, it is essential to note that despite their potential, studies exploring the use of supramolecular biopolymers for abiotic stress mitigation are relatively scarce. Further research is therefore necessary to understand their effectiveness in different abiotic stress conditions and their compatibility with diverse plant species.

### Supramolecular Biopolymers in Biosensors

Plant diseases offer considerable obstacles to worldwide agriculture, resulting in significant financial repercussions and jeopardizing the food supply’s stability. The timely identification and precise diagnosis of plant diseases are crucial for successfully implementing efficient disease management approaches. Conventional diagnostic approaches frequently depend on time-consuming techniques, imposing constraints on their feasibility to monitor diseases on a broad scale. Hence, an urgent need exists for novel sensing and diagnostic methodologies that afford expeditious, highly responsive, and discerning identification of plant pathogens [137]. Over time, several screening approaches have been devised to detect and diagnose various illnesses resulting from pathogenic agents.

The standard analytical detection methods for plant pathogens have been classified into distinct categories, including direct and indirect procedures. The direct approaches encompass two specific techniques namely Polymerase Chain Reaction (PCR) and detection of volatile organic chemicals (VOCs) emitted by the infected plants. In instances when there is a need for high selectivity and sensitivity, nucleic acid-based detection methods are employed, including destructive analysis conducted within a laboratory setting subsequent to the sample extraction. The increasing prevalence of biosensors has demonstrated their efficacy in on-site applications, offering enhanced sensitivity, mobility, and time efficiency [137]. Biosensors have been developed as potent instruments for detecting plant diseases due to their notable attributes of high sensitivity, selectivity, and quick response [138]; they afford a detectable signal from chemical reactions by incorporating a biologically active component and suitable transducer [139]. Biosensors comprise a physiochemical transducer and a molecular recognition element, referred to as a bioreceptor molecule, which interacts with specific target analytes. The bioreceptor encompasses diverse biological components, including but not enzymes, DNA, antibodies, entire cells, tissues, and organelles. The production of biorecognition information occurs when receptors engage with the target analytes. The transducer then transforms this information into various forms, such as electrochemical, electrical, optical, or thermal signals [40, 137, 140].

In recent decades, increasing evidence has demonstrated the feasibility of employing biosensing approaches to detect plant pathogens. These techniques have shown promising potential in delivering meaningful diagnostic outcomes in many practical settings. Khater et al. [138] and Patel et al. [137] have presented a comprehensive overview of several biosensing methodologies used in identifying and detecting *Pythium* sp., the causal agent of damping-off. A biosensor was synthesized utilizing cellulose films wherein it has been suggested that this approach for detecting phytopathogenic pythium could be applied to surveillance plant diseases within the agricultural sector [141]. Also, Regiart et al. devised an electrochemical immunosensor to facilitate the timely identification of *Xanthomonas arboricola* in walnut plant samples. The diagnosis exhibited a threefold increase in speed compared to (enzyme-linked immunosorbent assay) ELISA, while also demonstrating considerably enhanced specificity and sensitivity [142]. Biosensors consist of various components: (1) To detect and respond to analyte, a bioreceptor could come as a nucleic acid, enzyme, antibody, gene, or a whole cell or orange [143]. (2) Various immobilization procedures are employed to link bioreceptors with base materials [144]. (3) Base materials (transducer surface) commonly comprise several substances, such as metals, glass, or composites [145–147].

**Fig. 6:**
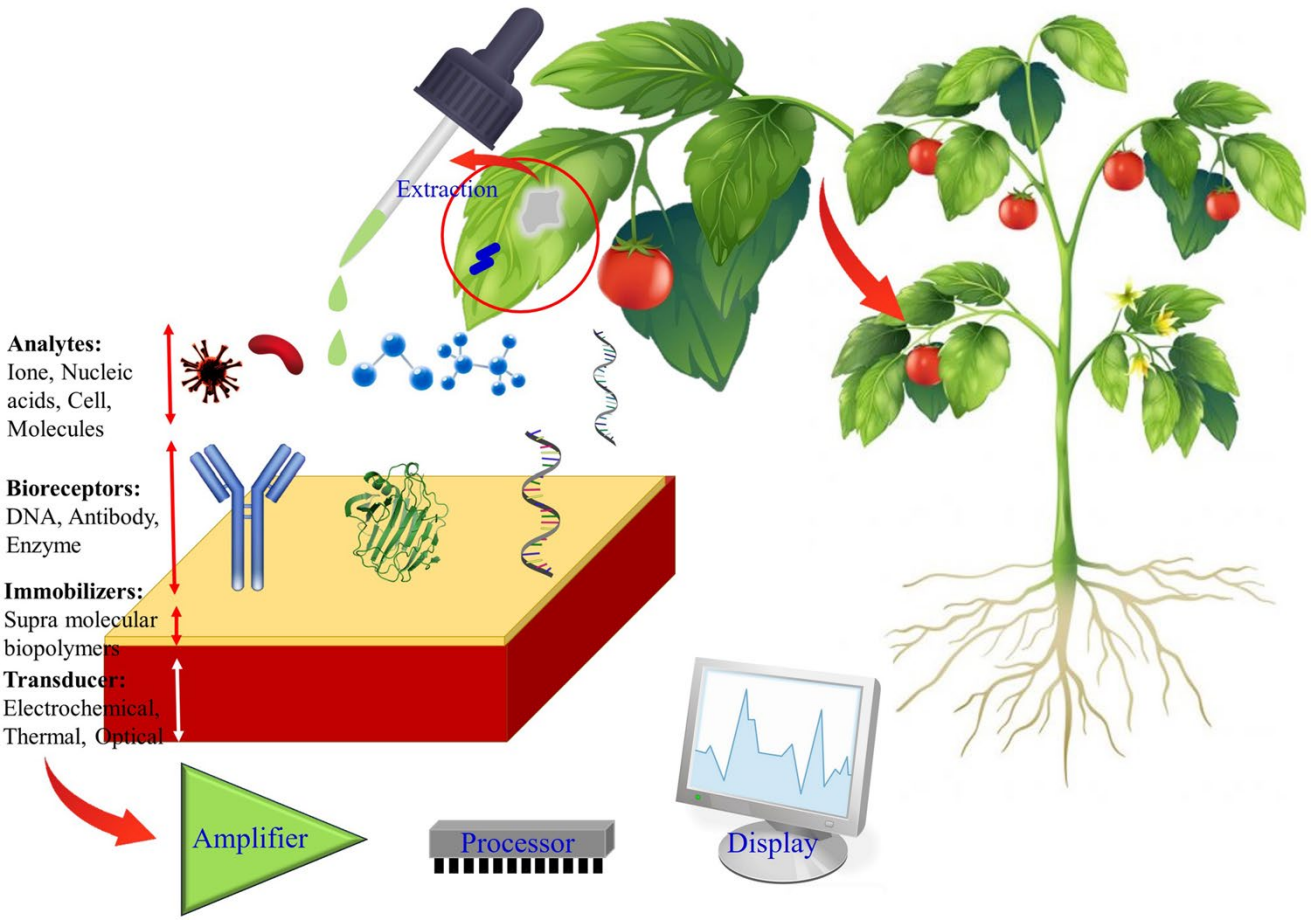
Schematic components of biosensors and the application of supramolecular polymer in their structure as immobilizers

The effective creation of a biosensor relies on the connection between the biorecognition unit and the analyte. The immobilization and stabilization of the biological treatments on the transducer’s surface affect several aspects of the sensor’s performance, including the degree of its sensitivity, and selectivity [40]. One of the primary obstacles in advancing biosensors is the necessity to immobilize and stabilize the bioreceptors on the base materials, a process that significantly impact the sensor’s sensitivity, selectivity, and stability [148, 149]. A potential solution to this challenge involves applying nano/micro-structural supramolecular biopolymers as a coating on the transducer surface (base material). This approach enables the bioreceptors’ immobilization on the base materials, improve the biosensor’s performance, and mitigates non-specific interactions with the sample matrix [150]. Figure 6 depicts a schematic of biosensor components and the application of supramolecular polymer in their structure as an immobilizer.

The use of nano/micro-structural supramolecular biopolymers as a coating on transducer surfaces offers several advantages over conventional polymers, including (1) the assembly of supramolecular biopolymers at the nanoscale has the potential to enhance the surface area, thereby offering advantageous prospects for application in catalysis or sensing [151]; (2) supramolecular biopolymers are synthesized via non-covalent interactions, encompassing hydrogen bonding, electrostatic interactions, and Van der Waals forces. The mechanism described enables the polymer structure to undergo dynamic and reversible assembly, disassembly, and rearrangement in response to various environmental stimuli, including changes in pH, temperature, or the presence of other molecules [152]; (3) supramolecular biopolymers possess the ability to self-heal, thereby repairing any damage to their structure without requiring external intervention. The aforementioned characteristic is significant in biosensors, where the biopolymer is susceptible to mechanical impairments [153].

Several studies have presented conclusions on the creation and use of nano/micro-structural supramolecular biopolymers in the diagnostics context. Qin et al. developed a polymer composite of sodium alginate and poly(diallyl dimethyl ammonium chloride), which exhibited electrostatic interaction and was then used to cast a film onto a gold electrode. Their findings demonstrated that the analytical efficacy of these supramolecular biopolymers displayed superior characteristics compared to biosensors that utilize conventional biopolymers, such as polysaccharides, as matrices for enzyme immobilization [104]. Also, Hue, et al. applied a matrix based on Co^2+^ and supramolecular polymer to immobilize glucose oxidase for enzymatic glucose sensing [105]. In this context, other documented evidences have also confirmed that these biopolymers can be utilized to synthesize biosensors as superior biosensors.

Although limited information is available on applying supramolecular biopolymers to detect plant pathogens, the research conclusions suggest that these polymer-based biosensors can be as effective and superior tools to immobilize biological elements on the transducer’s surface.

## Challenges and Future Prospectives

In applying nano/micro-structural supramolecular biopolymers in agriculture, several current limitations and obstacles need to be addressed. One major challenge is the cost associated with producing and processing these materials, as the manufacturing processes tend to be expensive, limiting their accessibility to farmers, particularly in developing countries. To overcome this limitation, it is crucial to have a resolved focus on cost-effectiveness and identifying pathways to make these entities affordable. Another obstacle is the scalability of production. Scaling up the manufacturing processes to meet the demand of large-scale agricultural operations is necessary for widespread implementation. It is essential to optimize production techniques to ensure efficient and scalable manufacturing without compromising quality. The stability of these polymers is another concern as they need to withstand environmental conditions such as extreme temperature, UV radiation, and microbial degradation. Ensuring their stability over extended periods in various agricultural environments is essential to maximize their effectiveness.

Aside from the technological difficulties, the use of nano/micro-structure supramolecular biopolymers in agriculture ought to be considered ethically. In this regard, a comprehensive evaluation of the safety and possible effects on human health and the environment is required when new materials are introduced into the agriculture industry. It is necessary to establish or update regulatory frameworks in order to assure the conscientious and sustainable use of nano/micro-structured supramolecular biopolymers in agricultural contexts. This entails evaluating their enduring impacts on soil health, water quality, and biodiversity, while also defining protocols for appropriate use and disposal techniques. Additionally, collaborative endeavors among academics, policymakers, industrial stakeholders, and agricultural communities are important to advance the secure and morally sound deployment of these biopolymers. Adopting a multidisciplinary approach helps guarantee that regulatory frameworks and standards are based on scientific data, social values, and the practical requirements of farmers. It is necessary to create educational and awareness initiatives to educate farmers, agricultural practitioners, and the general public about the advantages, hazards, and correct application of nano/micro-structured supramolecular biopolymers. Facilitating the appropriate adoption of these novel materials may be achieved by promoting responsible stewardship and offering resources for training and technical assistance.

Further research and development can address these limitations and pave the way for advancement with a focus on optimizing the performance and functionality of nano/micro-structural supramolecular biopolymers. Improving their mechanical strength, water absorption capacity, controlled release properties, and stability under different environmental conditions will enhance their effectiveness in agricultural applications. Tailoring these biopolymers to specific crops and soil types are another avenue for research. Understanding their interaction with different crop species and soil compositions will enable targeted and effective applications. Moreover, integration with precision agriculture and smart farming technologies offers exciting possibilities. Combining nano/micro-structural supramolecular biopolymers with sensors, data analytics, and automated systems can optimize their usage and application efficiency. Nano/micro-structural supramolecular biopolymers enhance the sustainability aspects by being non-toxic, biodegradable, and eco-friendly. Their application reduces the need for conventional pesticides and fertilizers, thus promoting safer farming practices and minimizing environmental contamination. These biopolymers also have the potential to build climate-resilient agricultural practices. By improving soil structure, water management, and nutrient efficiency, they help farmers adapt to changing climate conditions and enhance crop resilience. Therefore, despite current limitations, nano/micro-structural supramolecular biopolymers hold great promise for the future of agriculture. Overcoming cost barriers, optimizing performance, and integration with emerging technologies will drive their widespread adoption. Implementing these biopolymers can lead to sustainable farming practices that prioritize resource efficiency, reduce chemical inputs, and enhance climate resilience.

## Conclusion

Nano/micro-structured supramolecular biopolymers have gained significant attention in the agricultural industry due to their non-toxicity, biodegradability, self-assembly, adaptability, and biocompatibility. These biopolymers offer tailored solutions to various agricultural challenges, such as improving plant growth and development, increasing soil fertility and structure, enhancing water retention, and promoting crop resilience in dry areas. One of the key advantages of these biopolymers is their ability to release nutrients and agrochemicals in a controlled manner, reducing environmental pollution and optimizing nutrient absorption by plants. They also function as intelligent delivery systems, allowing precise administration of bioactive substances to targeted sites, thereby enhancing effectiveness, and minimizing unintended impacts. Supramolecular biopolymers can also be used in targeted formulations to activate defense chemicals and cell walls, triggering specific signaling pathways against infections and pests. Additionally, they show promise as biosensors for detecting phytopathogens by immobilizing biological components on transducer surfaces. Nevertheless, additional investigations are warranted to fully understand the potential of biopolymers in agriculture as these structures offer sustainable and efficient farming methods that ensure food security, protect the environment, and improve agricultural systems.
